# The Effect of Lanthanum (III) Nitrate on the Osteogenic Differentiation of Mice Bone Marrow Stromal Cells

**DOI:** 10.1007/s12011-023-03723-y

**Published:** 2023-06-19

**Authors:** Qian Wang, Yi-Fan Li, Hai-Song Zhang, Xue-Zhong Li, Yan Gao, Xing Fan

**Affiliations:** 1 Division of Nephrology, Affiliated Hospital of Hebei University, No. 212 of Yuhua East Road, Lianchi District, Baoding, 071000 Hebei China; 2 Key Laboratory of Bone Metabolism and Physiology in Chronic Kidney Disease of Hebei Province, No. 212 of Yuhua East Road, Lianchi District, Baoding, Hebei 071000 China; 3 Department of Integrated Chinese and Western Medicine, Affiliated Hospital of Hebei University, No. 212 of Yuhua East Road, Lianchi District, Baoding, Hebei 071000 China; 4 Intensive Care Unit, Affiliated Hospital of Hebei University, No. 212 of Yuhua East Road, Lianchi District, Baoding, Hebei 071000 China

**Keywords:** Lanthanum nitrate, Bone marrow stromal cells, Osteogenic differentiation, Lanthanum-containing precipitation

## Abstract

To study the species of lanthanum (III) nitrate (La[NO_3_]_3_) dispersed in cell media and the effect on the osteoblast differentiation of bone marrow stroma cells (BMSCs). Different La-containing precipitations were obtained by adding various concentrations of La(NO_3_)_3_ solutions to Dulbecco’s modified Eagle medium (DMEM) or DMEM with fetal bovine serum (FBS). A series of characterisation methods, including dynamic light scattering, scanning electron microscopy, transmission electron microscopy, energy-dispersive X-ray spectroscopy, and protein quantification were employed to clarify the species of the different La-containing precipitations. The primary BMSCs were isolated, and the cell viability, alkaline phosphatase activity, and the formation of a mineralised nodule of BMSCs were tested when treated with different La-containing precipitations. The La(NO_3_)_3_ solutions in DMEM could form LaPO_4_, which exits in the particle formation, while the La(NO_3_)_3_ solutions in DMEM with FBS could form a La-PO_4_-protein compound. When treated with La(NO_3_)_3_ solutions in DMEM, the cell viability of the BMSCs was inhibited at the concentrations of 1, 10, and 100 μM at 1 day and 3 days. Meanwhile, the supernatant derived from the La(NO_3_)_3_ solutions in DMEM did not affect the cell viability of the BMSCs. In addition, the precipitate derived from the La(NO_3_)_3_ solutions in DMEM added to the complete medium inhibited the cell viability of the BMSCs at concentrations of 10 μM and 100 μM. When treated with La(NO_3_)_3_ solutions in DMEM with FBS, the derived precipitate and supernatant did not affect the cell viability of the BMSCs, except for the concentration of 100 μM La(NO_3_)_3_. The La-PO_4_-protein formed from the La(NO_3_)_3_ solutions in DMEM with FBS inhibited the osteoblast differentiation of BMSCs at the concentration of 1 μM La(NO_3_)_3_ (*P* < 0.05) but had no effect on either the osteoblast differentiation at the concentrations of 0.001 and 0.1 μM or on the formation of a mineralised nodule at all tested concentrations of La(NO_3_)_3_. Overall, La(NO_3_)_3_ solutions in different cell culture media could form different La-containing compounds: La-PO_4_ particles (in DMEM) and a La-PO_4_-protein compound (in DMEM with FBS). The different La-containing compounds caused different effects on the cell viability, osteoblast differentiation, and the formation of a mineralised nodule of the BMSCs. The La-containing precipitation inhibited the osteoblast differentiation by inhibiting the expression of osteoblast-related genes and proteins, providing a theoretical basis for clinical doctors to apply phosphorus-lowering drugs such as lanthanum carbon.

## Introduction

With the progression of chronic kidney disease, disorders of phosphate metabolism can emerge [1]. Hyperphosphatemia, a common complication in patients with end-stage renal disease (ESRD), is closely associated with the occurrence of renal osteopathy and cardiovascular calcification [1, 2]. Such a disease not only seriously damages the quality of life in patients, but also increases the incidence of cardiovascular diseases and mortality [1, 2]. The treatment methods for hyperphosphatemia involve dietary restriction, dialysis, and oral phosphate binders [3]. However, strict management of a low-phosphate diet can lead to malnutrition, and normal dialysis can only modestly reduce the serum phosphate levels [3, 4]. Therefore, the application of phosphate binders presents an effective clinical approach for hyperphosphatemia [2, 3].

In 2004, lanthanum carbonate (La_2_ [CO_3_]_3_) (Fosrenol) was approved for the treatment of hyperphosphatemia in patients with ESRD in the USA and Europe, largely because it has a higher efficacy and tolerability compared to other conventional phosphate binders [5]. After La_2_(CO_3_)_3_ is taken orally with meals, La^3+^ is released in the gastrointestinal tract and binds with dietary phosphate to produce La phosphate, which is excreted via the faeces. In humans, La_2_(CO_3_)_3_ is understood to be barely absorbed in the gastrointestinal tract [6–8]. However, two Japanese groups reported that La can be absorbed in the gastrointestinal mucosa, particularly in the stomach, and transported via the lymph flow of patients treated with La_2_(CO_3_)_3_ [9–11]. Due to the similarity of La and calcium in terms of physical and chemical properties, the former has been recognised as a ‘bone-seeking’ element [12]. The question thus remains as to how the absorbed La affects the bone metabolism.

Previous studies found that La may interfere with the bone-remodelling process and affect the function of bone cells (13). Liu et al. [13] found that lanthanum chloride (LaCl_3_) has two opposing effects on the proliferation, osteogenic differentiation, and mineralisation of MC3T3-E1 cells, significantly increasing these processes at lower concentrations and inhibiting the processes at higher concentrations. Elsewhere, Xu et al. [14] reported that La^3+^ inhibits the differentiation of bone marrow stromal cells (BMSCs) into osteoblasts in the early stage via activation of a mitogen-activated protein kinase signalling pathway, while Jiang et al. [15] demonstrated that LaCl_3_ inhibits osteoclast formation, function and osteoclast-specific gene expression in vitro. However, the absorbed La may form precipitates in biological systems [14, 16]. While the effects of La^3+^ on bone cells have been previously reported, the biological effects of La-containing precipitates are not well understood [14].

Bone marrow stroma cells have the potential to differentiate into many types of cells, such as osteoblasts, adipose cells, and fibroblasts, under certain conditions [17]. They thus present an excellent cell differentiation model [17]. In this study, the biological effects of La-containing precipitates on the proliferation, osteogenic differentiation, and mineralisation of BMSCs at the cellular and molecular levels are investigated for the first time. The aim of this research was to study the species of La(NO_3_)_3_ dispersed in cell media and to assess the effect on the osteoblast differentiation of BMSCs, which will provide guidance for the application of La_2_(CO_3_)_3_ drugs.

## Materials and Methods

### Materials

Dulbecco’s modified Eagle’s medium (DMEM) and fetal bovine serum (FBS) were purchased from Gibco (Thermo Fisher Scientific, Rockville, MD, USA). Lanthanum nitrate hexahydrate (purity 99.999%), 3-(4,5-Dimethylthiazol-2-yl)-2, 5-diphenyltetrazolium bromide (MTT), dimethyl sulfoxide (DMSO), benzylpenicillin, streptomycin, dexamethasone, ascorbic acid, β-glycerophosphate, and alizarin red S (ARS) were purchased from Sigma-Aldrich (St. Louis, MO, USA). An alkaline phosphatase (ALP) activity kit and a Coomassie brilliant blue protein assay kit were obtained from Nanjing Jiancheng Biological Engineering Institute (Nanjing, China).

### Isolation and Culture of Bone Marrow Stroma Cells

The BMSCs were prepared from 4–6-week-old specific pathogen-free (SPF) Institute of Cancer Research mice (Beijing Vital River Laboratory Animal Technology Company, Beijing, China) following the method devised by Maridas D.E et al. [18]. In brief, the mice were reared adaptively under standard conditions for a week and then executed via cervical vertebra. The animal experimental operations were performed in accordance with the Guide for Animal Care and Use of Affiliated Hospital of Hebei University, with the animal experiments approved by the Ethics Committees and Health Authorities of Affiliated Hospital of Hebei University (No. IACUC-2021003SM). Femora and tibiae were harvested under aseptic conditions, and the whole bone marrow was flushed using supplemented DMEM in a 10-cc syringe and a 25-gauge needle. The BMSCs were collected and cultured in DMEM with 12% FBS, 100 U/mL streptomycin, and 100 U/ml penicillin for 3 days in a carbon dioxide (CO_2_) incubator (5%CO_2_, 95% air; Model MCO-18AIC, Sanyo, Osaka, Japan,) at 37 °C. The culture medium was replaced every 3 days during the experiments.

### Particle Size Measurement

The gradient concentrations of lanthanum (III) nitrate (La[NO_3_]_3_) (0.001, 0.01, 0.1, 1, 10, 100 μM; Sigma) were freshly prepared in DMEM with or without 10% FBS. The mixture was then vortexed for 5 s before being subjected to sonication in a water bath at room temperature with a power of 120 W for 60 s to reduce the agglomeration. The particle size distribution was measured using a Zetasizer (Nano-ZS90; Malvern Panalytical, Malvern, UK) The above solution was filtered through a 0.22-μm filter, and the size distribution of the filtrate was measured using the above method.

### Electron Microscopy and Energy Dispersive X-Ray Analyses

The morphology of the particles was evaluated via scanning electron microscopy (SEM) (JSM-7500F; Japan Electronics Co., Ltd, Tokyo, Japan) and transmission electron microscopy (TEM) (Tecnai G2 F20 S-TWIN, Field Electron and Ion Company, Hillsboro, TX, USA). In the presence of cells, the morphology and the location of the particles formed in 10% FBS-supplemented DMEM or serum-free DMEM were observed via SEM. The BMSCs (3 × 10^6^ cells per well) were plated in 24-well culture plates and cultured at 37 °C in a 5% CO_2_ humidified incubator before being exposed to 100 μM La(NO_3_)_3_ in DMEM with or without 10% FBS and to 10% FBS-supplemented DMEM without La for 6 h. The cells were fixed with 3% glutaraldehyde and precooled at 4 °C followed by fixation with 1% osmium tetroxide. Following dehydration in a series of gradient ethanol, the samples were sputtered with gold and observed via SEM. The element content in the particles deposited on the cells was determined using an energy dispersive X-ray (EDX) microanalyzer (proX).

### Cell Viability Assay

The cell viability was determined using a modified version of Mosmann’s method based on the cleavage of the MTT, with the research procedures performed as previously described [19]. Briefly, the BMSCs were plated in 96-well culture plates with a density of 3 × 10^6^ cells per well and cultured at 37 °C in a 5%CO_2_ humidified incubator. Then, the cells were exposed to various concentrations of La(NO_3_)_3_ in gradient doses of 0, 0.001, 0.01, 0.1, 1, 10, and 100 μM in DMEM with or without 10% FBS. Following incubation for 24 and 72 h, the MTT dye solution (10 μL, 5 mg/mL; Sigma) was added. The mixture was then cultured for 4 h in the incubator. Following this, the supernatant was removed, and 100 μL of DMSO was added. The absorbance was detected using a microplate spectrophotometer (MD VersaMax, Molecular Devices, LLC., San Jose, CA, USA) at a wavelength of 570 nm. To clarify the contribution of the particles to the cell viability, a series of filtration experiments were performed. Compared to that of the filtrate, which was obtained by filtering the La-containing medium through a 0.22-μm filter, the effect of serum-free DMEM and 10% FBS-supplemented DMEM-containing La at gradient concentrations of 0, 0.001, 0.01, 0.1, 1, 10, and 100 μM was investigated. The BMSCs were incubated with the unfiltered medium and the filtrates, respectively. As noted above, the cell viability (%) was evaluated using the MTT method and was calculated according to the following formula:

$$\left({OD}_{treated}-{OD}_{blank}\right)/\left({OD}_{control}-{OD}_{blank}\right)\times 100$$

### Alkaline Phosphatase Activity Assay

The research procedures were performed following the method devised by Mohammed et al. [20]. The BMSCs (3 × 10^6^ cells per well) were seeded in 48-well plates with the osteogenic induction supplement (OS) containing 0.1 mol/L β-glycerophosphate, 0.5 g/L ascorbic acid, and 10^−7^ mol/L dexamethasone [15]. La(NO_3_)_3_ in gradient doses of 0, 0.001, 0.01, 0.1, and 1 μM were added to the culture medium and then cultured at 37 °C in a 5%CO_2_ humidified incubator for 10 days. The BMSCs treated with OS only were set as the control group. Then, the BMSCs were washed twice with ice-cold phosphate-buffered saline (PBS; Sigma) following incubation before the Triton-X-100 solution (120 μL, 1%; Sigma) was added. After a 30-min incubation, the BMSCs were lysed completely. The ALP activity and protein content were evaluated using the ALP activity kit (Nanjing Jiancheng Biological Engineering Institute, Nanjing, China; No. A059-2) and a micro-Bradford assay kit(Nanjing Jiancheng Biological Engineering Institute, Nanjing, China; No. W042-1), respectively. All results were standardised according to the protein content.

### Mineralised Matrix Formation Assay

The BMSCs (3 × 10^6^ cells per well) were seeded in 24-well culture plates with the OS. La(NO_3_)_3_ in gradient doses of 0, 0.001, 0.01, 0.1, and 1 μM were added to the culture medium and then cultured at 37 °C in a 5%CO_2_ humidified incubator for 21 days. The BMSCs treated with OS only were set as the control group. The formation of mineralised matrix nodules was determined via ARS staining. In brief, the cells were fixed in 95% ethanol for 10 min at room temperature. The fixed cells were then washed with ice-cold PBS and stained with ARS (200 μL, 0.1%; Sigma) for 30 min at 37 °C in a 5%CO_2_ humidified incubator. Then, the plates were washed with ice-cold PBS again and photographed using a microscope (Olympus, Tokyo, Japan; BX-53). Quantitative analysis of the ARS staining was performed via elution with 10% (w/v) cetylpyridiniumchloride for 20 min at room temperature, with the optical density (OD) detected at a wavelength of 570 nm. The mineralisation promotion rate (%) was analysed according to the following formula:

$$\left({OD}_{treated}-{OD}_{blank}\right)/\left({OD}_{control}-{OD}_{blank}\right)\times 100$$

### The Quantitative Reverse Transcription-Polymerase Chain Reaction Analysis

In the presence of OS, the total ribonucleic acid (RNA) from the BMSCs treated with 0.001 and 1 μM La(NO_3_)_3_ for 10 days was prepared using Qiagen RNeasy Mini kits (Qiagen; No.74106) before being reversely transcribed into first-strand complementary DNA (cDNA) according to the TransScript protocol. Quantitative reverse transcription-polymerase chain reaction analysis (qRT-PCR) was conducted in a total volume of 20 μL with 2 μL cDNA, 4 μL of gene-specific PCR primer pair stock, and 10.4 μL of SYBR® Green/ROX Master Mix (Vazyme; No.Q311-02) using an ABI 7000 Sequence Detection System. To activate TaqDNA polymerase, the PCR profiling commenced with 10 min at 95 °C, followed by 40 cycles of 30 s at 95 °C, and 60 s at 60 °C, and then 30 s at 72 °C. The relative amount of messenger RNA expression standardised to glyceraldehyde-3-phosphate dehydrogenase (GAPDH) was expressed in terms of fold change, which was analysed using the comparative CT (2^−ΔΔCt^) related to the control group as a reference (2^−ΔΔCt^ = 1). The relative primers involved in the qRT-PCR analysis are shown in Table 1.

**Table 1:** The qRT-PCR primers

Gene name	Forward primer (5′-3′)	Reverse primer (5′-3′)
OPG	AGTCCGTGAAGCAGGAGTG	CCATCTGGACATTTTTTGCAAA
BMP-2	TGGCCCATTTAGAGGAGAACC	AGGCATGATAGCCCGGAGG
OCN	GAACAGACTCCGGCGCTA	AGGGAGGATCAAGTCCCG
GAPDH	GACTTCAACAGCAACTCCCAC	TCCACCACCCTGTTGCTGTA

### Statistical Analyses

The data were collected and presented in terms of mean ± the standard deviation. The statistical analyses were performed using a Student’s *t-*test, with *P* < 0.05 indicating statistical significance.

## Results

### Particle Size Distribution of Lanthanum (III) Nitrate in Different Media

**Fig. 1:**
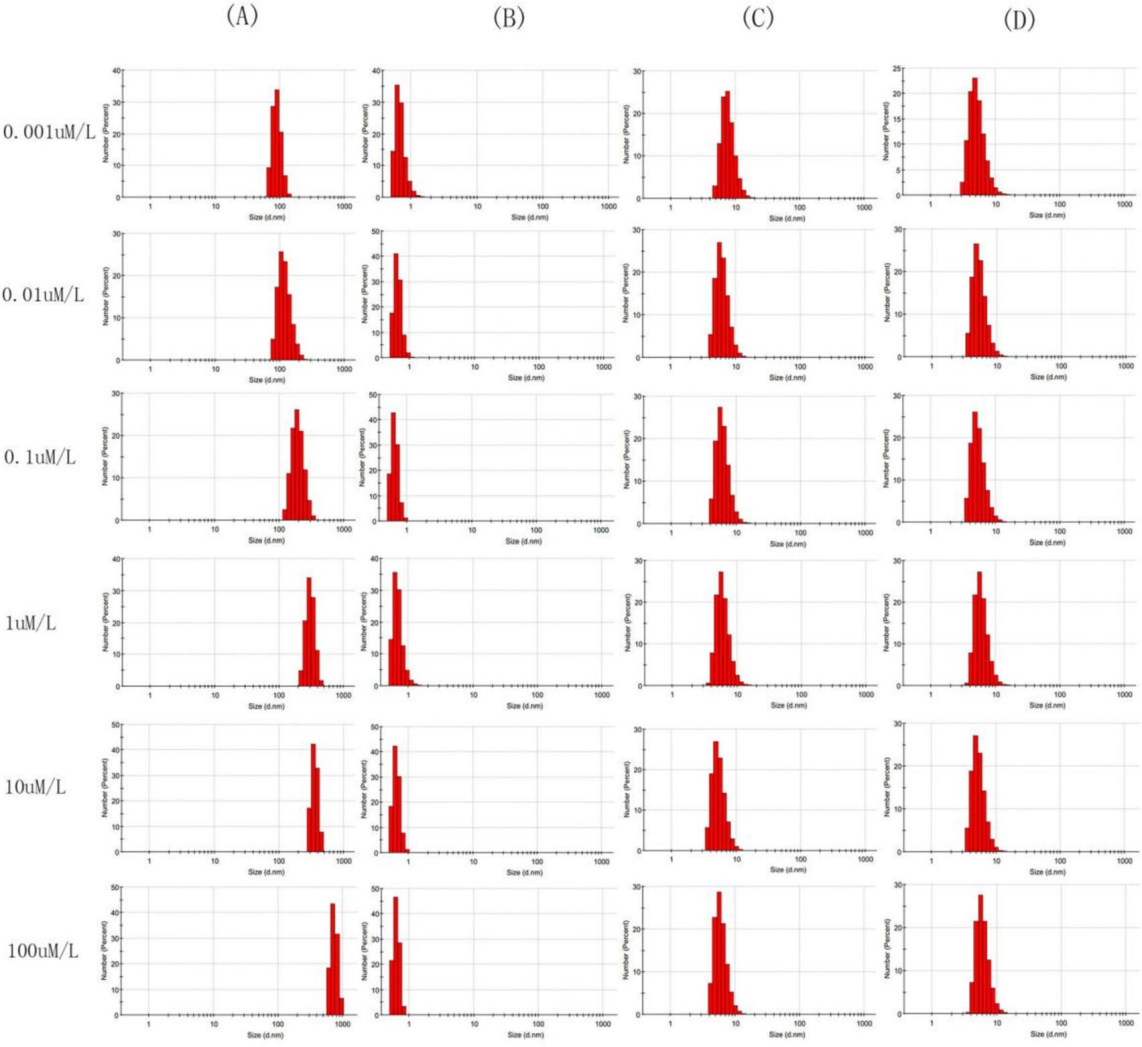
Particle size distribution of different media containing La(NO_3_)_3_ and filtrates after filtration. **A** Particle size distribution of serum-free DMEM containing different concentrations of La(NO_3_)_3_. **B** Particle size distribution of the filtrate after the DMEM containing different concentrations of La(NO3)3 without FBS passed through the 0.22-μm filtration membrane. **C** Particle size distribution of DMEM containing 10%FBS with different concentrations of La(NO_3_)_3_. **D** Particle size distribution of the filtrate after the DMEM containing 10%FBS with different concentrations of La(NO_3_)_3_ passed through the 0.22-μm filtration membrane

When the DMEM solutions were treated with gradient concentrations of La(NO_3_)_3_, a fluffy precipitate instantly formed in the medium. Using dynamic light scattering (DLS) experiments, the particle size of the precipitates formed in serum-free DMEM was found to range from 91.16 to 746.2 nm on average, increasing with the increase in La(NO_3_)_3_ concentration (Fig. 1A). After filtering the aforementioned mixtures using a 0.22-μm filter, the particle size of the serum-free DMEM clearly decreased following the filtration (Fig. 1B). However, the particle size of the precipitates formed in 10% FBS-supplemented DMEM was found to be approximately 7 nm, regardless of La(NO_3_)_3_ concentration (Fig. 1C), while the 10% FBS-supplemented DMEM underwent no change after filtering the aforementioned mixtures using a 0.22-μm filter (Fig. 1D). This demonstrated that the La(NO_3_)_3_ formed different particles of varying sizes in the culture media with or without serum.

### The Morphology and Elemental Composition of the La-Containing Particles in Different Media

**Fig. 2:**
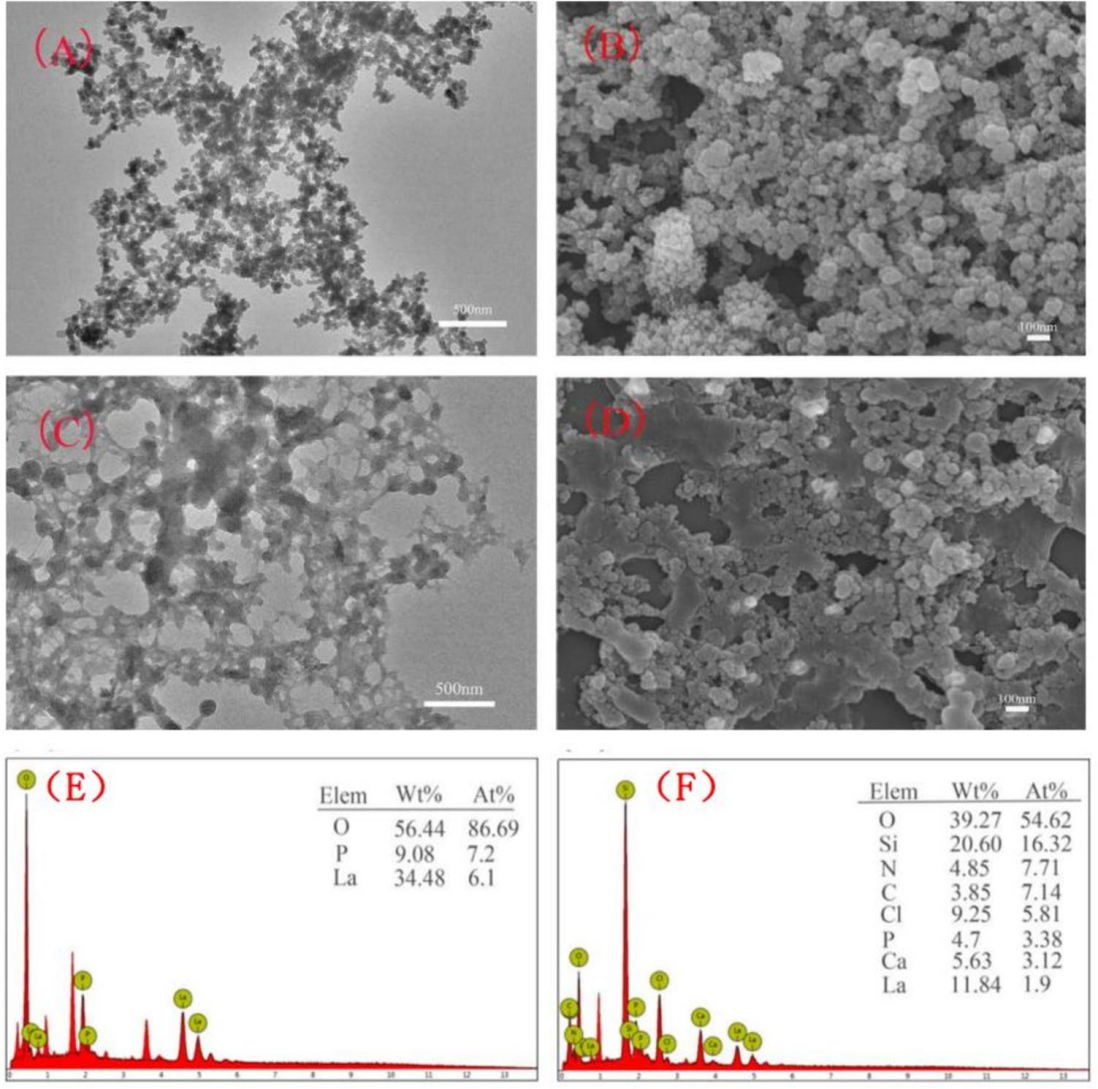
The TEM, SEM, and EDS images of precipitates formed by La(NO_3_)_3_ in different media. **A** The TEM image of the precipitate formed in DMEM without FBS at 50 μM La(NO3)3. **B** The SEM image of the precipitate formed in DMEM without FBS at 50 μM La(NO3)3. **C** The TEM image of the precipitate formed in DMEM containing 10%FBS at 50 μM La(NO3)3. **D** The SEM image of the precipitate formed in DMEM containing 10%FBS at 50 μM La(NO3)3. **E** The EDS spectrum of the precipitate formed in DMEM without FBS at 50 μM La(NO_3_)_3._ **F** The EDS spectrum of the precipitate formed in DMEM containing 10%FBS at 50 μM La(NO_3_)_3_

The morphology of the particles was further evaluated via SEM and TEM after blending the serum-free DMEM or 10%FBS-supplemented DMEM with 50 μM La(NO_3_)_3_. The La(NO3)3 formed spherical particles with a relatively uniform morphology in the serum-free DMEM, with the particles exhibiting a certain degree of agglomeration (Fig. 2A and B). However, the morphology of the precipitate formed in the medium changed considerably in the presence of the serum, and the precipitate formed in the 10%FBS-supplemented DMEM consisted of a larger-sized sheet and smaller-sized nanoparticles (Fig. 2C and D). The attendant elemental compositions were then evaluated via energy-dispersive X-ray spectroscopy (EDS), and the results for the particles formed in the serum-free DMEM indicated the mass% of the compounds is 32%, and the presence of La, phosphorous, and oxygen (Fig. 2E). As such, it can be inferred that the precipitate was composed of a La phosphate complex. However, in addition to the above elements, the elemental analysis of the precipitates formed in 10% FBS-supplemented DMEM indicated the presence of other elements, including carbon, nitrogen, and chlorine (Fig. 2F). It can thus be proposed that certain substances in the serum, such as proteins, are also involved in the formation of precipitates.

To confirm whether the precipitate formed in the 10% FBS-supplemented DMEM contained protein components, a series of protein quantitative tests were performed. As shown in Table 2, the precipitate formed in the 10% FBS-supplemented DMEM indeed contained protein components. This indicated that in the 10%FBS-supplemented DMEM containing La(NO_3_)_3_, the La possibly took the form of a La-PO_4_-protein compound.

**Table 2:** Protein quantification

Group	Protein content(gprot/L)		
	Complete medium	supernatant	precipitate
DMEM	0	–	–
10% FBS–DMEM	0.811	–	–
La(NO_3_)_3_ + DMEM	0	0	0
La(NO_3_)_3_ + 10% FBS–DMEM	0.889	0.832	0.076

### The Accumulated Ability of La-Containing Particles on the Surface of BMSCs

**Fig. 3:**
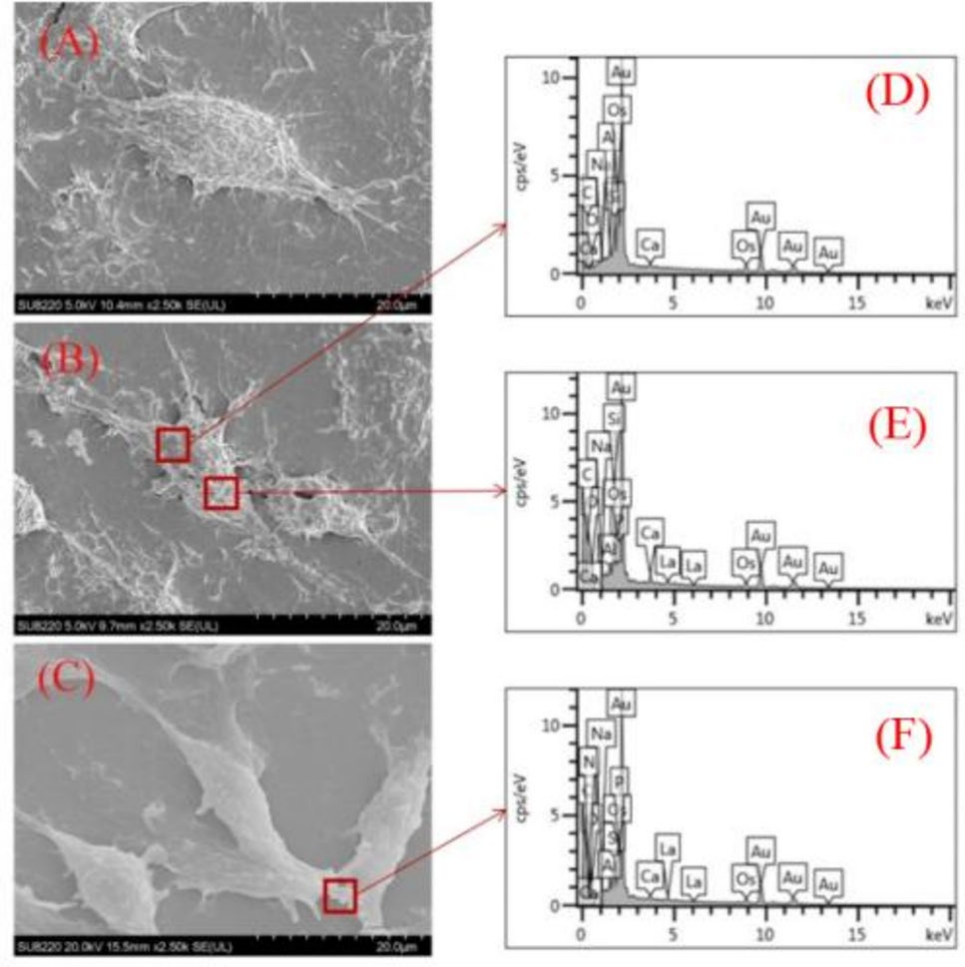
The SEM images of the precipitates formed by La(NO_3_)_3_ in the different culture media on the surface of the BMSCs and the elemental analysis results. **A** The SEM image of the BMSC surfaces of the control group. **B** The SEM image of the BMSC surfaces of the DMEM without FBS at 100 μM La(NO_3_)_3_. **C** The SEM image of the BMSC surfaces of the DMEM containing 10%FBS at 100 μM La(NO_3_)_3_. **D**–**F** The elemental analysis results

Furthermore, no particles were observed on the surface of the BMSCs without La(NO3)3 treatment (Fig. 3A). Conversely, the morphology and the location of the La-containing particles were evaluated via SEM after the incubation of the BMSCs in serum-free DMEM or 10% FBS-supplemented DMEM with 100 μM La(NO_3_)_3_ for 6 h. As shown in Fig. 3B and C, agglomerated particles gathered on the surface of the BMSCs. The EDS microanalysis of the deposits on the cellular surface (Fig. 3D–F) indicated that the mass% of the compounds is 32%, and La may has been deposited on the cell surface in the form of LaPO_4_-based particles in the serum-free DMEM or in the form of a La-PO_4_-protein compound in the 10%FBS-supplemented DMEM. This indicated that the La(NO_3_)_3_ formed the insoluble precipitate with the PO_4_^3−^ or protein in the culture medium, which was deposited on the cellular surface to exert the effect on the BMSCs.

### Effect of La-Containing Particles on the Proliferation of BMSCs

**Fig. 4:**
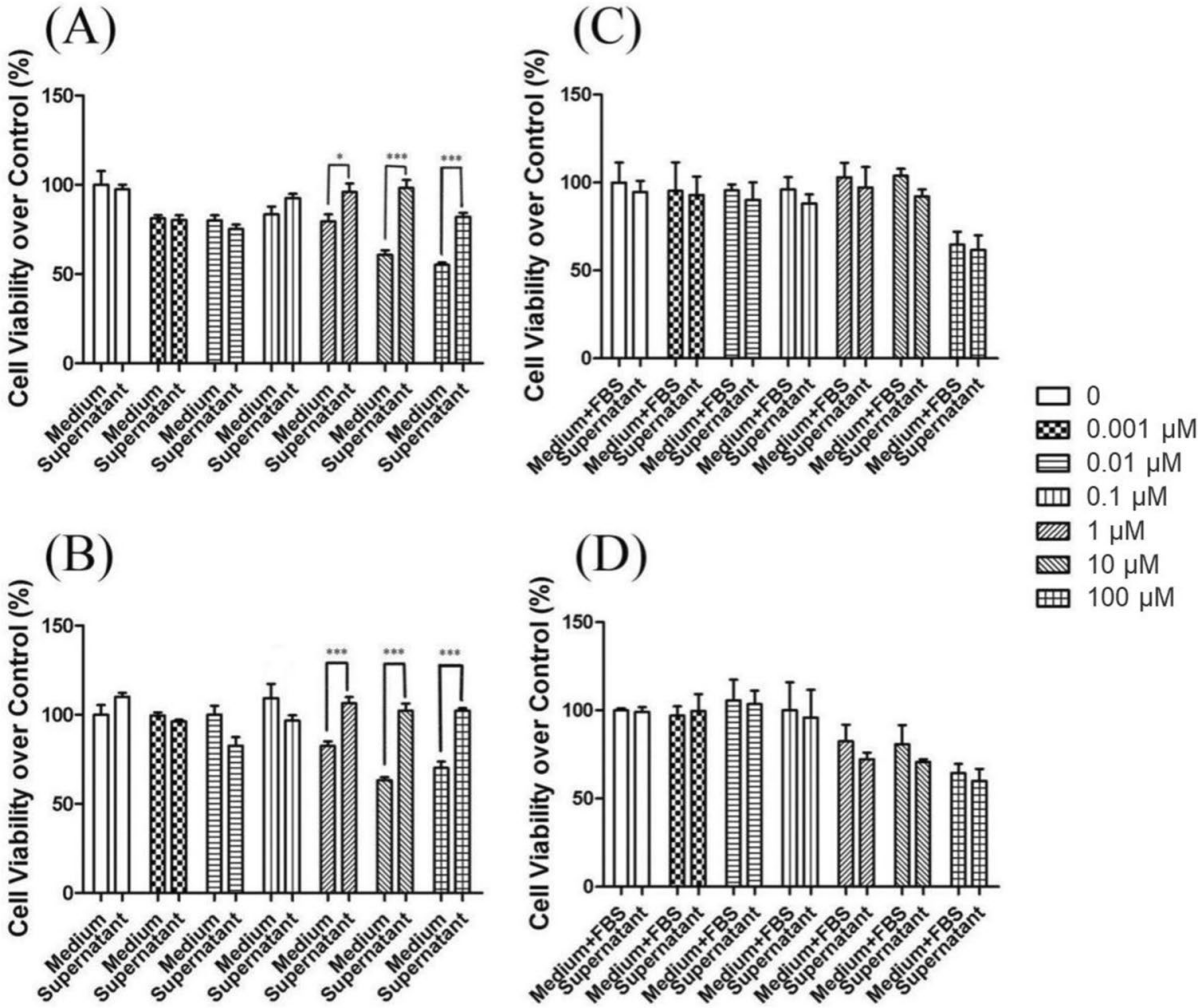
Effects of La.^3+^ and lanthanum-containing particles on BMSC cell viability. **A** and **B** Effects of FBS-free DMEM containing different concentrations of La(NO_3_)_3_ and filtrates over 0.22 μM on the cell viability of the BMSCs at 1 and 3 days. **C** and **D** Effects of DMEM–10%FBS containing different concentrations of La(NO_3_)_3_ and filtrates over 0.22 μm on the cell viability of the BMSCs at 1 and 3 days. *P < 0.05, ***P < 0.001 vs. control, *n* = 3

As shown in Fig. 4A–D, the effects of the La(NO_3_)_3_ on the proliferation of the BMSCs appeared in a concentration-dependent manner. After one and 3 days of La(NO_3_)_3_ treatment, the La(NO_3_)_3_ had no effect on the proliferation of BMSCs at gradient concentrations of 0.001, 0.01, 0.1, 1, and 10 μM, while it inhibited the proliferation of BMSCs at the concentration of 100 μM. The question thus remained as to whether the La-containing particles or the La^3+^ played the main role in BMSC proliferation. As such, the effects of the filtered and unfiltered media on the cell viability of BMSCs were compared. As shown in Fig. 4A and B, in the serum-free DMEM, the filtrate had no statistically significant effect. Conversely, the unfiltered parts decreased the cell viability of the BMSCs as the concentration of La(NO_3_)_3_ increased. However, in the 10%FBS-supplemented DMEM, there was no difference between the filtered and unfiltered media in terms of the cell viability of the BMSCs (Fig. 4C and D).

### Effect of La-Containing Particles on the Osteogenic Differentiation of BMSCs

**Fig. 5:**
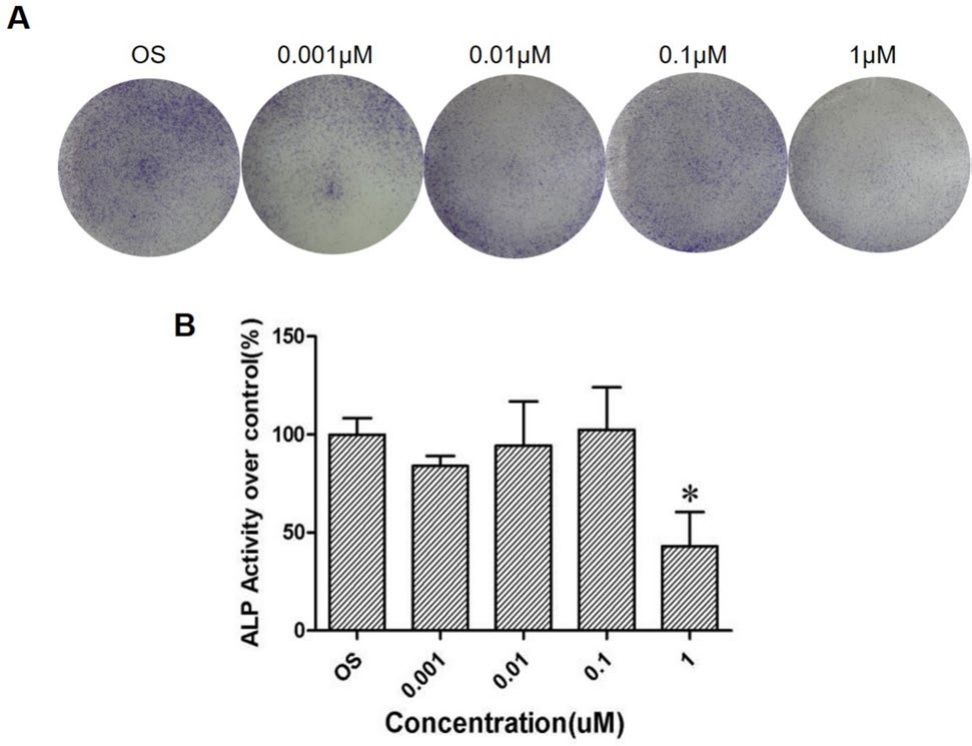
Effect of lanthanum-containing particles on ALP activity of BMSCs. **A** Representative images of ALP immunofluorescence. **B** The quantified analysis of lanthanum-containing particles on ALP activity of BMSCs that were treated with gradient doses of 0, 0.001, 0.01, 0.1, and 1 μM. *P < 0.05 vs. OS, *n* = 3

Images of the ALP staining results in the different groups are shown in Fig. 5A and B. After 10 days of La-containing particle treatment, it was found that the particles had no statistically significant effect on the ALP activity of the BMSCs at gradient concentrations of 0.001, 0.01, and 0.1 μM, while the ALP activity of the BMSCs was decreased at the concentration of 1 μM. This indicated that the La-containing particles inhibited the osteogenic differentiation of the BMSCs at high concentrations.

### Effect of La-Containing Particles on The mineralisation Ability of BMSCs

**Fig. 6:**
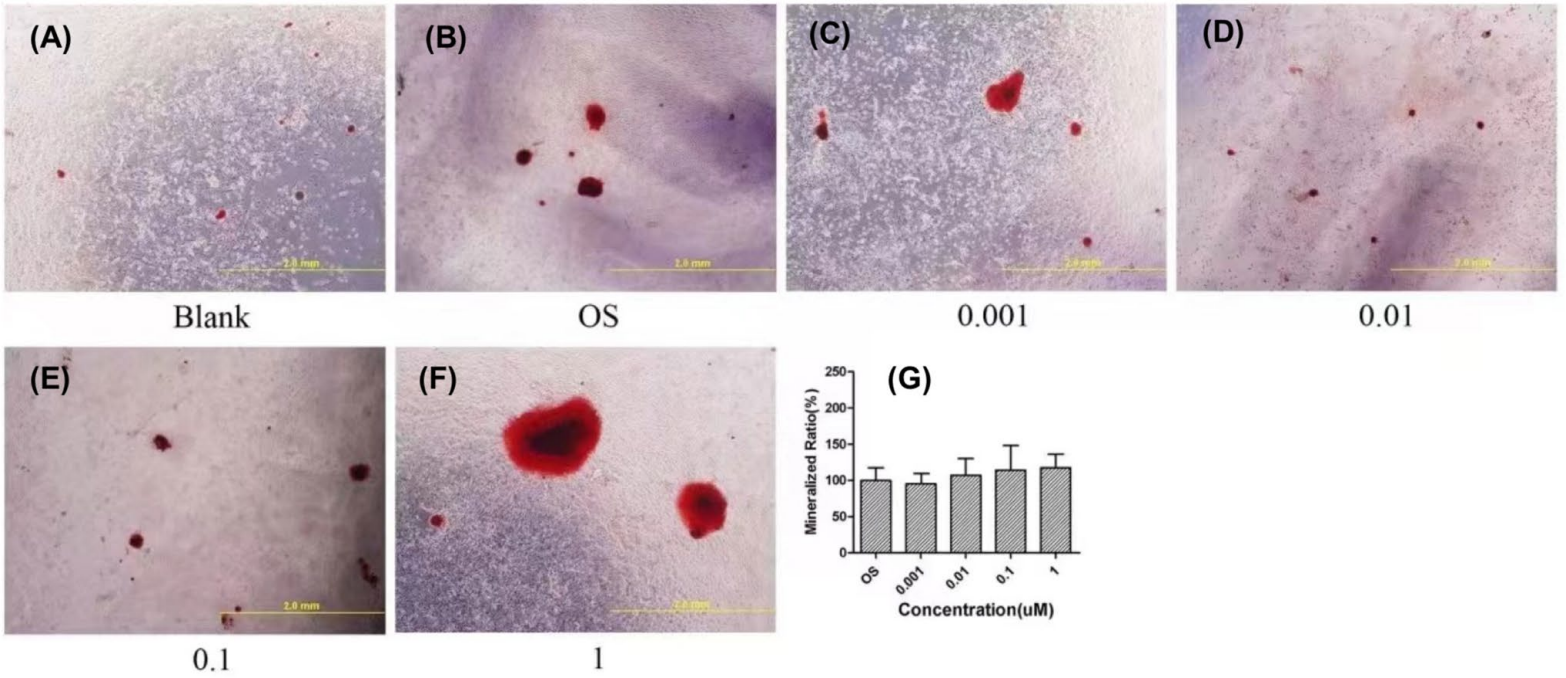
Effect of lanthanum-containing particles on mineralisation of BMSCs. **A**–**F** Representative images of the mineralised nodules of the BMSCs that were treated with gradient doses of 0, 0.001, 0.01, 0.1, and 1 μM, respectively. **G** The quantified analysis of lanthanum-containing particles on ALP activity of BMSCs that were treated with gradient doses of 0, 0.001, 0.01, 0.1, and 1 μM (*n* = 3)

As the morphological observation of the mineralised nodules indicated (Fig. 6A–F), at the concentrations of 0.001, 0.01, and 0.1 μM, the La(NO_3_)_3_ had no significant effect on the formation of mineralised nodules compared to the OS control group but promoted the formation of mineralised nodules at the concentration of 1 μM. The quantitative mineralisation data analysis also reflected this trend, but the difference was not statistically significant compared to the OS control group (Fig. 6G).

### The Inhibitory Effect of La-Containing Particles on the Expression of Osteoblast-Related Genes in BMSCs

**Fig. 7:**
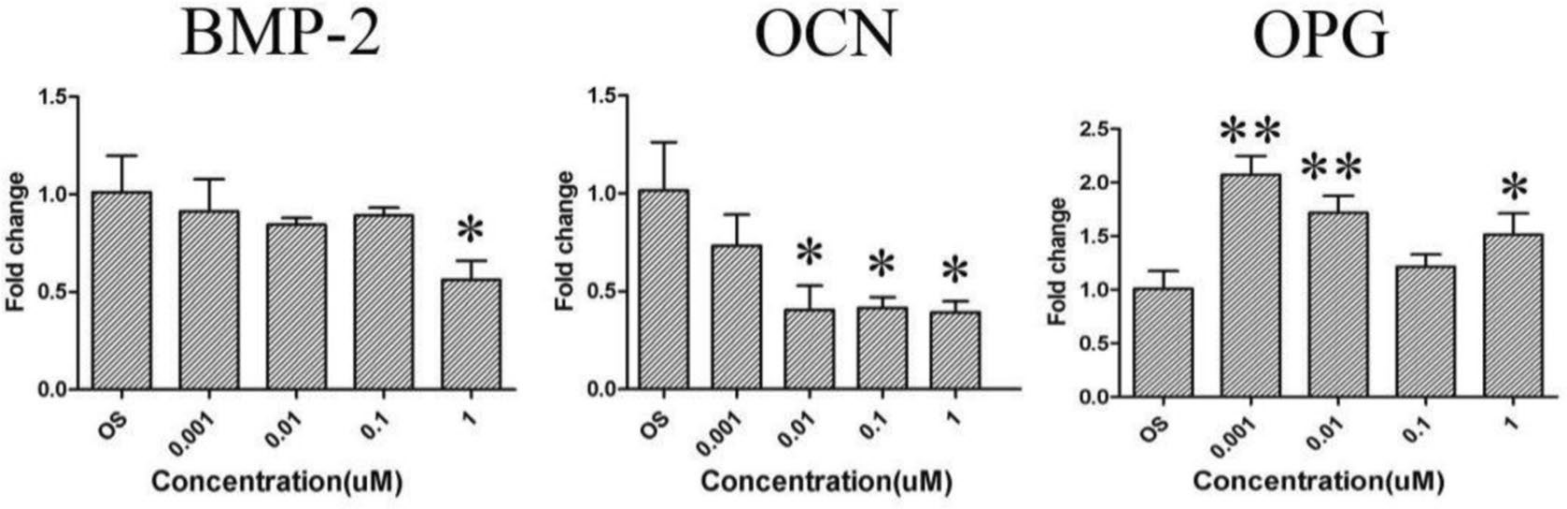
Effect of lanthanum-containing particles on the expression of osteoblast genes in BMSCs. *P < 0.05, **P < 0.01 vs. OS (*n* = 3)

The qRT-PCR method was used to detect the expression of osteoblast-related genes, with the results shown in Fig. 7. Here, the La(NO_3_)_3_ inhibited the expression of Bone morphogenetic protein-2 (BMP-2) and osteocalcin (OCN) and thus inhibited the osteoblast differentiation of the cells when the concentration was 1 μM. However, all concentrations of La(NO_3_)_3_ promoted the expression of osteoprotegerin (OPG).

## Discussion

Bone marrow stroma cells are the precursor stem cells of osteoblasts and adipocytes, and their differentiation plays a vital role in bone metabolism [21]. The regulation mechanism of the osteogenic and adipogenic differentiation of BMSCs is the major topic of current exploration [17]. Inhibiting the adipogenic differentiation of BMSCs and promoting the osteogenic differentiation to modulate the bone metabolism presents a new direction for the management of bone diseases [22]. Previous studies have shown that La^3+^ inhibits the differentiation of bone marrow stromal cells into osteoblasts in the early stage, by activating mitogen-activated protein kinase signalling pathways. The experimental conditions for these studies are all in cell-culture medium, which may lead to the formation of La-containing precipitates. Researchers often focus on the mechanism and potential pathways of its biological effects on cells, rather than the varieties of La^3+^ that produce the effects; thereby, our aim is to further investigate whether the biological effects of La on cells are due to La^3+^ or La-containing precipitates [14]. Hence, the relevant research on the influence of various La(NO_3_)_3_ solutions on the osteoblast differentiation of BMSCs will provide guidance for the application of La_2_ (CO_3_)_3_ drugs [5].

To date, LaPO_4_ nanoparticles have been widely used as imaging agents, drug carriers, and biomarkers [23]. In this study, it was first found that the La(NO_3_)_3_ solutions in different cell culture media formed different La-containing compounds: LaPO4 particles (in the DMEM medium) and a La-PO_4_-protein compound (in the DMEM medium with FBS). Furthermore, it was confirmed that La-containing particles accumulated on the surface of the BMSCs. Peng et al. [24] confirmed that La-MCS biocompatible components contribute to the adhesion, spreading, and proliferation of rat BMSCs. In addition, in the present study, it was found that La(NO_3_)_3_ could inhibit the proliferation of BMSCs in a concentration-dependent manner. The DLS results (Fig. 1) indicated that the particle size of the La-containing particles formed in serum-free DMEM was increased as the concentration of La(NO_3_)_3_ increased. The filtrate did not contain La since the larger particles could not pass through the 0.22-μm filter. However, the amount of La in the filtrate did not change since the 0.22-μm filter did not work on the smalicroler La-containing particles formed in the 10%FBS-supplemented DMEM. This demonstrated that the effect of La on the BMSCs pertained to the action of the La-containing particles formed in the media and not the La ions.

As an ectoenzyme, ALP acts as a marker for cells undergoing differentiation to form pre-osteoblasts and osteoblasts [25]. In general, the activities of ALP are increased following osteogenic induction in vitro for 7 days [25]. In addition, it was found that the La-containing particles inhibited the osteogenic differentiation of the BMSCs at high concentrations.

The formation of mineralised nodules is an indicator of advanced osteogenic differentiation. The influence of La-containing particles on the mineralisation ability of BMSCs was evaluated by detecting the mineralised nodules [26]. Here, it was found that La-containing particle treatment has no significant effect on the mineralisation of BMSCs. It should be noted that the 50 μM La(NO_3_)_3_ solution (Fig. 2) was selected to clearly show the morphology of the precipitate formed by La(NO_3_)_3_ in DMEM, while the doses in the other experiments were diluted in gradient proportion, with the concentration of 50-μM not incorporated in these experiments. In addition, a concentration of 100 μM is too high, potentially causing severe cell death, while a concentration of 10 μM is too low to have any effect. Thus, the effect of these concentrations on BMSCs was not investigated, with the appropriate mid-level concentrations selected for the experiments shown in Figs. 5 and 6.

Chu et al. [4] revealed that La-LDH scaffolds significantly suppress RANKL-induced osteoclastogenesis by inhibiting the NF-κB signalling pathway. Compared to scaffolds without La^3+^ dopants, La-LDH scaffolds provide a more favourable microenvironment to induce new bone in-growth along the icroporous channels [4]. Here, the La-containing particles inhibited the expression of osteoblast-related genes but enhanced the level of OPG in the BMSCs. In fact, OPG, a glycoprotein secreted by osteoblasts, can inhibit the formation and differentiation of osteoclasts [27]. The formation of bone entails a dynamic balanced process between osteogenesis and osteoclast [27]. The La-containing particles had no effect on the final mineralisation of the bone, which may have been related to the increased expression level of OPG. This finding was in line with that obtained by Chu et al*.* [4]. This study was conducted at the cellular level, and the results will be verified in vivo in future studies.

## Conclusion

This paper investigated the effect of precipitates formed by La(NO_3_)_3_ solutions in different cell culture media on the osteoblast differentiation of BMSCs. In the 10%FBS-supplemented DMEM containing La(NO_3_)_3_, the La possibly took the form of a La-PO_4_-protein compound. The effect of La(NO_3_)_3_ on the BMSCs pertained to the La-containing precipitate and not the La^3+^. The La-containing precipitates formed by a high concentration of La(NO_3_)_3_ in the cell culture media inhibited the activity of the BMSCs and inhibited osteogenic differentiation but had no effect on the final mineralisation. The La-containing precipitates inhibited the osteoblast differentiation of the cells by inhibiting the expression of the osteoblast genes, BMP-2, and OCN, but promoted the expression of the OPG gene, which may have been related to the final mineralisation osteogenesis. However, the molecular mechanism was only preliminarily discussed, and its potential signalling pathway merits further study. This article investigates the effect of La-containing particles formed by La(NO_3_)_3_ in cell culture medium on the osteogenic differentiation of BMSCs cells and its molecular mechanism, providing a theoretical basis for clinical doctors to apply phosphorus-lowering drugs such as lanthanum carbon.

## Data Availability

The datasets used and analysed during the current study are available from the corresponding author on reasonable request.
